# Correcting misperceived norms: An integrated intervention to prevent adolescent sexual violence and HIV in South Africa

**DOI:** 10.1080/17441692.2025.2593789

**Published:** 2025-12-04

**Authors:** Haley Adrian, Caroline Kuo, Akhona Rasmeni, Nandipha Gana, Lindsay M. Orchowski, Alan D. Berkowitz, Abigail Harrison, Yandisa Sikweyiya, Catherine Mathews

**Affiliations:** a Department of Health Studies, American University, Washington, D.C, USA; b The Department of Psychiatry and Mental Health, University of Cape Town, Cape Town, South Africa; c Health Systems Research Unit, South African Medical Research Council, Cape Town, South Africa; d Rhode Island Hospital, Alpert Medical School of Brown University, Providence, USA; e Independent Researcher and Practitioner, Mount Shasta, CA, USA; f School of Public Health, Brown University, Providence, USA; g Gender and Health Research Unit, South African Medical Research Council, Pretoria, South Africa; h School of Public Health, Faculty of Health Sciences, University of the Witwatersrand, Johannesburg, South Africa

**Keywords:** Adolescent health, sexual violence, HIV, social norms, South Africa, Gender equality, good health and well-being, reduced inequalities

## Abstract

South Africa is a critical hotspot in the global fight against sexual violence and HIV. We report on the development of *Schools Championing Safe South Africa*, a behavioural intervention that engages adolescent boys and their peers to identify and address misperceived norms related to these epidemics within the school setting. A social norms survey conducted among 1,431 students aged 13–19 at 3 high schools guided the development of intervention content. The survey captured self-reported and perceived peer sexual violence and HIV norms and behaviours. Analyses identified major misalignment (>20%) between perceived peer behaviours/norms and actual behaviours/norms. Perpetration of unwanted sexual petting, oral, vaginal, and anal sex was high: 61%, 53%, 53%, and 44% among boys, and 42%, 26%, 20%, and 18% among girls. We identified underestimation of peer support for bystander intervention, overestimation of peer acceptance of gender-based violence, and underestimation of the extent to which peers would believe a survivor. No misalignment between self and peer HIV risk behaviours were identified. Gaps between actual and perceived behaviours/norms are important targets to correct in a behavioural intervention. Given the interconnected risk factors associated with sexual violence and HIV, addressing them together presents a crucial opportunity to maximize prevention efforts.

## Introduction

South Africa sits at the global epicenter of sexual violence and HIV epidemics and is an ideal site to develop prevention science for adolescents most at risk for these urgent public health problems (Govender, 2023; Milovanovic et al., 2021). The bi-directional relationship between sexual violence and HIV indicates the need for an integrated preventive intervention (Dunkle & Decker, 2013). Adolescents are at an ideal life course stage for prevention interventions as the adolescent years are often when sexual violence and risk behaviors relating to HIV infection first occur (Jewkes et al., 2006; Richter et al., 2015). Although all genders and age groups are at risk for perpetration of violence and HIV, in South Africa, a particularly high-risk group is boys/men. In this setting, the vast majority of sexual violence is perpetrated by boys/men with a large community-representative sample in South Africa reporting 1 in 3 men (31.9%) engaging in rape perpetration (Jewkes et al., 2011).

The link between sexual violence and HIV is particularly relevant in the South African context, where both epidemics are highly prevalent and often co-occur (Jewkes et al., 2010; Li et al., 2014). This overlap stems from a range of interconnected factors, including the high rate of HIV among sexually active youth (Shisana et al., 2014), the association between sexual coercion and condomless sex (Dunkle et al., 2004; Fox et al., 2007), and shared structural drivers such as gender inequality, poverty, and stigma (Gibbs et al., 2020; Jewkes et al., 2015). While the relationship between sexual violence and HIV may be less pronounced in some global settings, understanding this intersection is crucial in South Africa, where addressing one without the other risks overlooking a key pathway of vulnerability. This dual burden provides a strong rationale for integrated prevention efforts targeting both forms of risk simultaneously.

Adolescent boys between the ages of 15 to 17 years are at high risk for sexual violence perpetration and HIV. In a large longitudinal prospective cohort study of South African adolescents, the median age of penetrative sexual debut was 15 years with 38.2% of boys engaged in penetrative debut at this age (Richter et al., 2015). In a large community-representative sample in South Africa, the average age of first sexual violence perpetration was at 17 years of age (Jewkes et al., 2006). Alignment of age of sexual debut with age of debut of sexual violence perpetration underscores the need for interventions during adolescence.

### Terminology

In this article, the WHO definition of *sexual violence* is used. This definition states that ‘sexual violence is any sexual act, attempt to obtain a sexual act, or other act directed against a person’s sexuality using coercion, by any person regardless of their relationship to the victim, in any setting.’ (WHO, 2022).

We also refer to *bystander attitudes and behaviors*, which describe individuals’ beliefs about and willingness to intervene in situations where there is a risk of harm, such as potential or actual sexual violence (Powell, 2011). *Bystander intervention* refers to actions taken by someone who is not directly involved in a harmful situation to interrupt, prevent, or reduce the risk of violence (Banyard et al., 2004). These behaviors may include direct intervention, seeking help, or providing support to individuals involved (Berkowitz, 2009).

We also refer to *sexual or heavy petting*, defined following Koss’s framework as fondling, kissing, rubbing against another person’s private areas (such as the lips, breasts/chest, genitals, or buttocks), or removing some of their clothing.

### Inter-related HIV and sexual violence epidemics

The intricate interplay between sexual violence and HIV underscores the imperative for a holistic preventive intervention. Several systematic reviews and meta-analyzes identify causal and non-causal contextual factors linking sexual violence and HIV. Causal factors linking increased HIV risk with sexual violence include increased genital or anal tissue trauma associated with increased infection risk (Cheng et al., 2023). Non-causal factors include positive correlation between HIV infection and those who perpetrate sexual violence (Dunkle et al., 2004), and higher rates of HIV risk behaviors among sexual violence perpetrators including decreased condom use (Fox et al., 2007; Frye at al., 2007), concurrent and/or multiple sexual partners (Frye et al, 2007; Sa & Larsen, 2008), alcohol and substance use, and higher rates of HIV and other sexually transmitted infections (STIs) (Kouyoumdjian et al., 2012; Seth et al., 2013). HIV acquisition risk is significantly higher among individuals who have experienced sexual violence (Abrahams et al., 2021). For example, a global systematic review and meta-analysis of 28 studies with *N* = 331,468 women (including 4 South African studies), indicated that any type of intimate partner violence (IPV) was significantly associated with HIV infection (Li et al., 2014).

While sexual violence and HIV share overlapping structural risk factors in South Africa, they are also influenced by distinct proximal drivers. Sexual violence is often rooted in harmful gender norms, power inequities, and social acceptance of coercion (Heise, 2011; Jewkes et al., 2015) and is therefore suited to a social norms correction approach. In contrast, HIV risk—particularly among adolescents—is more commonly associated with gaps in knowledge, limited sexual health communication, low self-efficacy, and lack of skills around negotiation and consent (Pettifor et al., 2015; Toska et al., 2017).

Accordingly, our intervention integrates two distinct, theory-driven strategies to address these interconnected epidemics. Part 1 is grounded in social norms theory and aims to correct misperceived norms that may facilitate high-risk behaviors and inhibit bystander intervention to prevent sexual violence. Part 2 draws on the Information–Motivation–Behavioral Skills (IMB) model to reduce HIV risk by building adolescents’ knowledge, motivation, and behavioral skills—while also addressing harmful norms and behaviors that are accurately perceived. Our structural intervention approach targets peers in the school environment, who play an important role in shaping boys’ norms and behaviors around relationships, gender roles, sex and violence, and HIV.

### Social norms approach

A recent systematic review identified only eight interventions tested using randomized controlled designs to concomitantly address sexual violence and HIV risk among adolescents in Sub-Saharan Africa. Only one of these was in a school environment, and none utilized a corrective social norms approach as the main pathway for change to address sexual violence and HIV concurrently (Righi et al., 2019). This gap reflects a broader need for developmentally tailored programs in adolescence that address the complexity of sexual violence behaviors across diverse contexts and relationships, including school settings (Carmo et al., 2025).

Social norms are differentiated from personal attitudes in that they convey ideas about behavior that is (mis)perceived to be normal and socially accepted (Paluck, 2009). Correcting misperceived social norms is a specific approach to promoting behavior change, and this approach has shown promise in preventing sexual violence (Gidycz et al., 2011; Orchowski et al., 2023) and dating violence in other global settings (Taylor et al., 2013). Social norms create a potentially powerful risk or protective pathway for interlinked risks of sexual violence and HIV (Orchowski & Berkowitz, 2022a, Orchowski & Berkowitz, 2022b). Correcting misperceived social norms and behaviors can promote positive preventive behaviors relating to reduction of sexual violence and prevention of HIV infection under four theoretical assumptions (Berkowitz, 2003, Berkowitz, 2010). These include the assumptions that: 1) community norms influence behavior; 2) positive community norms aligned with prevention can be reinforced; 3) community norms are often misperceived (i.e. they are over- or under-estimated) and misperceptions encourage individuals to adjust their attitudes and behaviors to conform to what they incorrectly perceive to be true; and 4) correcting misperceptions allows individuals to act in accordance with their actual beliefs, which are most often positive and health promoting (Perkins, 2003). Positive behavior is generally the norm, but individuals tend to believe that problem behavior is more common (Perkins et al., 2011). As such correcting misperceived norms can be an important pathway of change to behaviors relating to prevention of sexual violence and HIV (Lapinski & Rimal, 2005). However, it is important to recognize that unhealthy/negative social norms that are perceived correctly may the need for additional, complementary strategies to effectively address these social norms.

The social norms approach is ideally suited for the developmental stage of adolescence in well-defined communities such as schools. Early experiences around relationships, sexual behavior, and violence during adolescence can uniquely shape young people’s social norms around what is healthy/unhealthy norms for future relationships (Friedlander, 2007). Schools constitute important community environments for social norms formation; adolescents spend a large segment of their day in school and social interactions with student peers and teachers are critical to identity development and health behaviors (Duncan et al., 2001; Flood et al., 2009; Harris, 1998; Jacobson & Crockett, 2000; Rowe, 1994). Thus, leveraging the school ecology (specifically teachers and student peers) to establish healthy norms among boys just as they are establishing attitudes towards sex, relationships, and violence may help to shape patterns of healthy behavior across the lifespan.

### Objectives

This article reports on the development of intervention materials for *Schools Championing Safe South Africa*, a behavioral intervention that engages adolescent boys and their peers to address misperceived norms related to sexual violence and HIV within the school environment. To inform the intervention content, we conducted a social ecological survey among students (including the target population of the intervention, adolescent boys) at three South African high schools to assess norms, behaviors, and perceptions related to sexual violence and HIV.

The aims of this study were:

1. Part 1: To use survey findings to generate evidence-based social norms messages and develop a poster campaign designed to reveal and challenge misperceived norms and behaviors.
2. Part 2: To develop two sexual health curriculum sessions to help adolescents translate corrected norms and behaviors into motivation and behavioral practice for sustained preventive behavior change, and to begin addressing harmful norms and behaviors that were correctly perceived.

By assessing the broader school social environment and drawing on positive behaviors already practiced by many students—such as high rates of condom use—we aimed to develop content that could catalyze shifts in peer norms and support healthier behaviors among adolescent boys.

## Materials and methods

### Setting and participants

This study took place in three schools in the Western Cape of South Africa, within communities facing high rates of sexual violence and HIV. These schools are situated in densely populated urban townships predominantly inhabited by Xhosa-speaking residents.

We conducted a cross-sectional social ecological survey of 1,431 adolescents aged 13 to 19 years, in grades 8 through 12, between February and March 2022. Our goal was to reach 75% of enrolled students at each participating school in order to capture a representative sample of the school population. Although the behavioral intervention primarily targets boys aged 15–17, we included a broader sample of adolescents aged 13–19 of all genders, recognizing that boys’ behaviors related to violence and HIV are shaped by interactions with a wider peer group. Participants completed the survey following parental consent (for those under 18) and adolescent assent. As a token of appreciation, each participant received a small gift, such as a pen or pencil. Ethical approval was obtained from institutional review boards at Brown University and the South Africa Medical Research Council.

### Procedures

Each potentially eligible study participant was informed about the study by members of our research team during school hours. Participants were then sent home with parental/guardian consent forms and assent forms. For potential participants under 18 years of age, parents/guardians were given a minimum of 1 week to review forms, ask questions, and return forms. If adolescents returned forms that indicated parental consent for participation in the study, they moved on to the assent process which involved an interactive discussion with study staff about risks and benefits of participation. Adolescents who were 18 years of age or older provided signed informed consent before participation and adolescents who were under 18 years of age provided signed informed parental/guardian consent forms along with signed informed assent forms before participation. Participants were provided with an electronic survey on a tablet. The survey was answered on a tablet in privacy using audio-computer assisted self-interviewing where questions were both visually displayed and with question-and-answer response read aloud and heard through private headphones linked to the tablet (without interviewer administration) to diminish social desirability bias that might influence the honesty of answers to sensitive questions. The survey was conducted during a time allocated by the schools within the regular school day, chosen to ensure minimal disruption to students’ academic activities. The survey data was also gathered anonymously without any personal identifiers or participant research identifiers, to ensure that participants could answer as honestly as possible. All study materials and procedures were provided in both English and isiXhosa, the dominant language spoken in the study setting, and participants could refer to either or both.

### Development of intervention content

To inform the development of the Schools Championing Safe South Africa intervention, we first conducted a cross-sectional social ecological survey to assess students’ self-reported behaviors, perceived peer norms, and actual peer norms related to sexual violence and HIV. These data allowed us to identify school-specific misperceptions—particularly instances where pro-social attitudes and preventive behaviors were common but perceived to be rare.

#### Part 1 – Poster campaign (corrective social norms approach)

**Figure 1.**
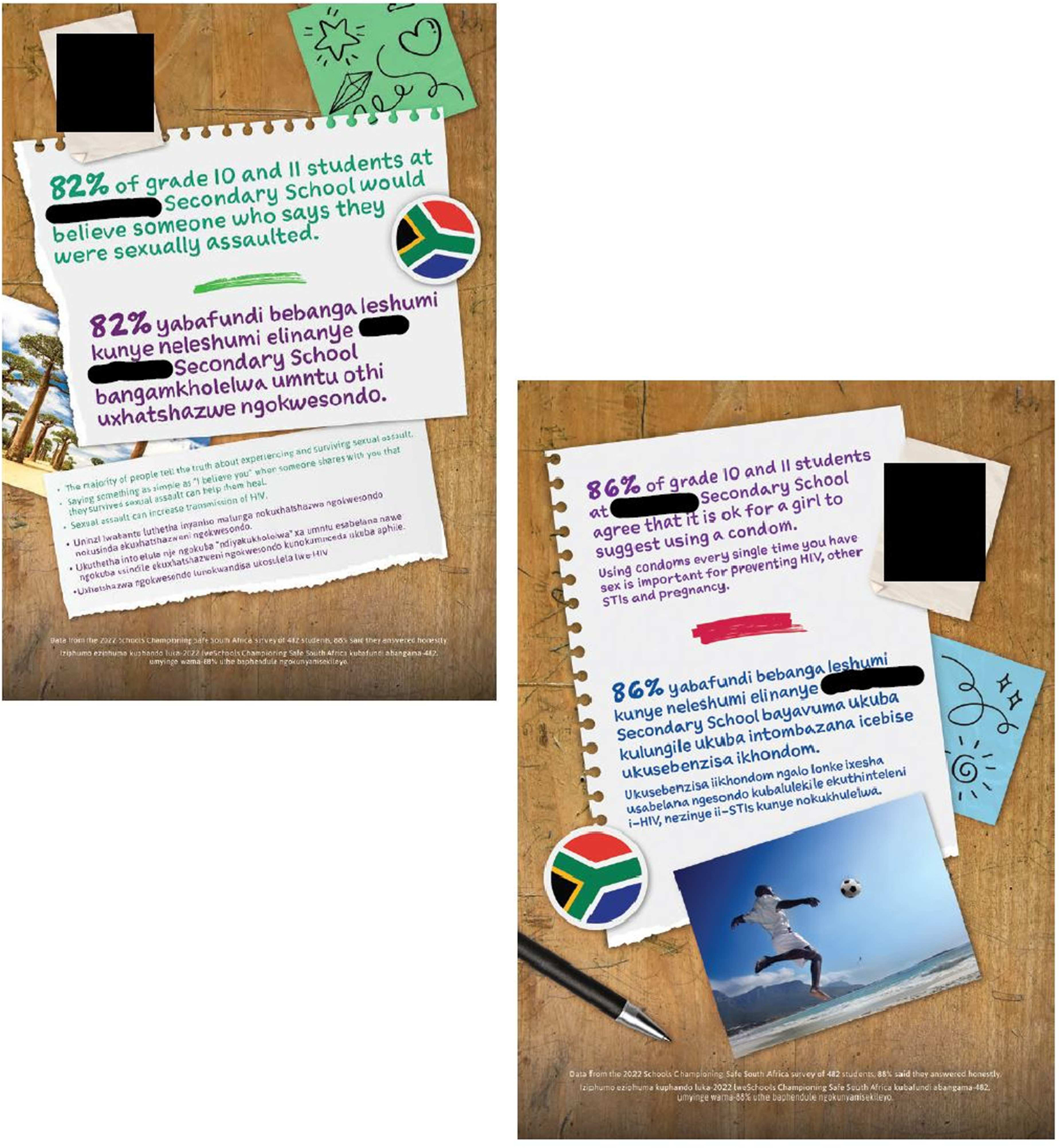
Examples of social norms posters featured at schools.

Survey analysis focused on identifying the largest gaps (≥20%) between actual and perceived peer norms/behaviors. Major misperceptions of pro-social norms (e.g. underestimating peer support for bystander intervention) and accurate perceptions of pro-prevention norms (e.g. high condom use) were prioritized for message development. Based on these findings, we drafted preliminary messages and refined them through focus groups with students and interviews with teachers at a refinement school. Feedback was used to select a unifying campaign theme, ensure content clarity, and optimize visual appeal. Twelve posters per school were produced, each linked to a specific misperception identified from that school’s survey data (see Figure 1). Posters were displayed in high-traffic areas and rotated every two weeks to avoid habituation. Each poster also displayed the percentage of students reporting they had completed the survey honestly to enhance credibility.

#### Part 2 – Curriculum sessions (IMB-based behavioral prevention approach)

Survey findings also identified harmful behaviors and attitudes that were not misperceived — for example, norms accurately perceived as inequitable or behaviors known to be high-risk. Because these are less amenable to change through a corrective norms approach alone, they were incorporated into two classroom-based sexual health curriculum sessions. These sessions aimed to increase knowledge, motivation, and behavioral skills for prevention, while also addressing these accurately perceived but harmful norms. Content was adapted from evidence-based sexual health education resources and tailored with examples and discussion topics from our survey results. Lessons included skill-building activities (e.g. condom negotiation role plays) and opportunities to discuss both positive and harmful norms.

Both components were implemented concurrently to reinforce key messages, with posters providing ongoing exposure and curriculum sessions offering deeper engagement and skills practice. This manuscript focuses on the development process; future publications will report on feasibility and acceptability.

### Measures

The social ecological survey assessed the following: (1) self-reported sexual violence and HIV risk behavior; (2) perception of their peers’ engagement in sexual violence and sexual behaviors in their school; (3) self-reported norms and attitudes around sex, sexual violence, gender roles, and HIV; and (4) perceptions of peer norms and attitudes around sex, sexual violence, gender roles, and HIV. All measures were offered in English or isiXhosa. All measures were designed for or tested in adolescent populations. For isiXhosa, all measures were translated into English and then back translated into isiXhosa. Below we briefly describe all measures that were used in our analyzes.

### Self-reported sexual violence behavior

Past 12-month completed and attempted sexual violence perpetration was assessed through the Sexual Experiences Survey—Short Form Perpetration focused on frequency of engaging in forced or coerced sexual petting, oral sex, vaginal sex, and anal sex (Koss et al., 2007). A sample item assessing completed acts included ‘How many times in the past 12 months did you fondle, kiss, or rub up against the private areas of someone’s body (lips, breast/chest, crotch or butt) or remove some of their clothes when they did not want this,’ with follow-up questions asking whether a coercion (e.g. ‘Was this by telling lies, threatening to end the relationship’) or force (i.e. ‘Was this by using force.’) tactic was used.

### Self-reported HIV risk behavior

Two initial items assessed lifetime history of engaging in vaginal and anal sex. Participants endorsing no lifetime sex history were gated out of subsequent questions assessing recent sexual activity. Two items assessed lifetime number of vaginal or anal sexual partners; multiple sex partners was defined as more than one sexual partner. The following question assessed lifetime condom use during anal or vaginal sex, ‘Have you ever used a male or female condom during sexual intercourse? By ‘use,’ we mean wearing a condom for the entire duration of anal or vaginal sex.’ One additional item assessed perceived risk for HIV from Low (0–10%) to Very High (above 60%).

### Self-reported norms and attitudes around sex, sexual violence, gender roles and HIV

The Sexual Social Norms Inventory (Bruner, 2002) examines normative attitudes and behaviors related to sexual assault. Participants self-report items along a 5-point scale. Internal consistency in the current sample was good (Cronbach’s *α* = .83). The Bystander Intervention subscale of the Sexual Social Norms Inventory (Bruner, 2002), which examines personal engagement in bystander behavior, was used to assess participant’s likelihood to intervene when witnessing inappropriate dating situations. Higher scores indicate a greater likelihood to intervene. Internal consistency in the current sample was excellent (Cronbach’s *α* = .89). Personal attitudes toward and acceptance of rape myths were assessed with an adaption of the Illinois Rape Myth Acceptance Scale (Cook-Craig et al., 2014). Participants responded along a 4-point scale (0 = strongly disagree to 4 = strongly agree) to indicate their agreement with statements such as: ‘Sexual assault charges are often used as a way of getting back at guys.’ Items were summed so that higher scores represented higher acceptance of rape myths.

### Perception of peer norms and attitudes around sex, sexual violence, gender roles, and HIV

The Differential Reinforcement subscale of the Social Norms Measure (Boeringer et al., 1991), assessed participant’s perception that their peers disapproved of sexual aggression. Higher scores indicate greater perceived peer disapproval of sexually aggressive behavior. Internal consistency in the current sample was good (Cronbach’s *α* = .82). To measure perceived peer engagement in bystander behavior, participants completed the Bystander Intervention subscale of the Sexual Social Norms Inventory (Bruner, 2002). Higher scores indicate greater perceived peer use of prosocial bystander behaviors. The Gender-Equitable Men Scale (GEMS; Pulerwitz & Barker, 2008), assessed the level to which participants perceived their peers to hold gender equitable or inequitable beliefs (e.g. ‘For most boys my age at my school, he would be outraged if his wife asked him to use a condom.’). Higher scores corresponded to more equitable gender norms. Internal consistency in the current sample was good for both the inequitable gender scale items (Cronbach’s *α* = .85) and the equitable gender scale items (Cronbach’s *α* = .87).

### Analysis

Analysis focused on identifying major misalignments between perceived peer behaviors and actual behaviors; perceived norms and actual norms. Major misalignment between peer behaviors/norms and actual behaviors/norms was operationalized as a calculated difference greater than or equal to 20%. There is no established threshold in misperceived social norms research for identifying a gap between perception and reality, but our team felt that 20% was an acceptable working threshold for this analysis. We did not run misperceived norm calculations on all variables from each of the measures described; instead, we analyzed a shortlist of variables identified through formative work with other studies conducted with similar populations in South Africa. This shortlist is presented in Table 2 in the Appendix, while the full measures with all variables are presented in Table 3.

Although our target population for the intervention is boys, we did not disaggregate perceived peer behaviors and actual behaviors, as well as perceived peer norms and actual norms by gender because all genders have an important influence on boys’ behavioral decisions around HIV and sexual violence.

Most data on individual and peer social norms/behaviors utilized measures that collected responses on a 4- or 6-point Likert scale. Other measures collected responses on the perception of peer norms/behaviors utilizing a percentage scale response (1–100%). Because response options varied among measures within the survey (i.e. Likert scale, percentage scale) we transformed the responses in a way that made them comparable. For all 4-point Likert scale questions, responses were dichotomized into agree (agree; strongly agree) and disagree (disagree; strongly disagree). For all 6-point Likert scale questions, responses were dichotomized into likely (somewhat likely; likely; moderately likely; very likely) and unlikely (slightly likely; not at all likely). Using these dichotomized response categories, we then calculated the percentage of participants in each category (agree, disagree; likely, unlikely). This process was replicated for each applicable question to make responses comparable to percentages. The specific measures and variables used to calculate these differences are detailed in Table 1.

To calculate the discrepancy between the actual and perceived norm/behavior, we subtracted the actual self-reported norm/behavior percentage from the perceived peer norm/behavior percentage. This allowed us to then identify misperceived norms/behaviors with a difference of over 20%.

All forms were checked for missing data in the field and during entry. Key variables were examined for skewness, variability, missing data, and outliers, with transformations to achieve normality, if needed.

## Results

### Sample characteristics

Ninety-eight percent of participants identified as Black African (a category defined by the South African census) and 95% spoke mostly isiXhosa at home. The mean age of participants was 16.5 years. Sixty two percent of participants identified as girls, 37% as boys, and 1% as non-binary. These results are also reflected in Table 1.

#### Part 1: Findings relevant to corrective social norms intervention

We analyzed the shortlisted variables drawn from the measures described in the Methods section to identify misperceptions between perceived peer behaviors/norms and actual behaviors/norms. Here, we present only those where core misperceived norms were identified. Table 2 presents all shortlisted variables used to calculate misperceived norms; those that appear in bold and italics represent major misperceptions consistently identified across all three schools.

Major misperceived norms were identified in three topic areas:

1. Bystander intervention to prevent sexual violence—On average, participants thought only 48% of students at their school believed that bystanders could take steps to prevent sexual violence, when in reality 79% of participants endorsed active bystander intervention.
2. Acceptability of gender violence—Forty-nine percent of participants thought that most boys at their school believed it was *not* okay to hit a woman if she cheated on him, when in reality 78% of participants said this behavior was not okay.
3. Believability of survivors of sexual violence—On average, participants thought that only 49% of students at their school would believe someone who said they were sexually assaulted, when in reality 83% said they would believe a survivor.

Ninety percent of participants reported that they answered the survey honestly.

While these misperceived norms formed the basis for Part 1 of the intervention (poster campaign), findings on correctly perceived but harmful norms and high-risk behaviors informed the content of Part 2 (curriculum sessions), which aimed to build knowledge, motivation, and behavioral skills to reduce HIV risk and address these entrenched norms.

#### Part 2: Findings relevant to curriculum-based behavioral prevention intervention

There was a high prevalence of sexual violence perpetration, and as expected, rates of completed acts of sexual violence were higher among boys than girls. Boys reported completing acts of unwanted sexual petting, oral sex, vaginal sex, and anal sex (61%, 53%, 53%, 44%, respectively). Girls reported completing acts of unwanted sexual petting, oral sex, vaginal sex, and anal sex (42%, 26%, 20%, 18%). (Boys and girls combined rates: 49% sexual petting, 36% oral, 32% vaginal, and 27% anal sex, respectively.)

Sixty-two percent of participants reported ever having vaginal sex, and 30% reported ever having anal sex. Of those who reported ever having sexual intercourse, 76% had ever used a male or female condom during anal and/or vaginal sex. Of those who had never had sex, 83% reported it was because they were afraid of HIV/AIDS. Twelve percent of participants self-reported high or very high perceived risk for contracting HIV.

We did not find major misalignments between perceived peer behaviors and actual behaviors relating to HIV protective behaviors. While there were high rates of sexual activity, there were also high rates of protective behavioral norms (e.g. condom use among those who had sex). Of those who reported having had sexual intercourse before (vaginal or anal), 29% reported having had more than three sexual partners in their lifetime.

We found that harmful norms around consent-related behaviors were perceived relatively accurately. On average, participants believed that only about half (51%) of students at their school were likely to stop sexual activity if a partner asked them to stop, even if the partner had initially agreed to it. Reflecting similar percentages, 57% of students reported that they themselves would be likely to stop under those circumstances. A similar pattern of accurately perceived but harmful norms was observed in areas related to gender equity.

Because these norms could not be corrected through a social norms campaign, they informed the design of curriculum sessions that emphasized skill-building and motivation to support topics related to respectful sexual decision-making, navigating various types of consent, gender-equitable attitudes, and healthy relationship behaviors.

## Discussion

### Main findings

This study described the process of developing a school-based intervention—comprising a poster campaign and two classroom sessions—to address adolescent sexual violence and HIV risk behavior in South Africa. Rather than testing causal relationships, we interpreted descriptive patterns through established theory to guide the design of intervention content.

Findings revealed a high prevalence of sexual violence perpetration and HIV risk behaviors among adolescents, underscoring the need for integrated prevention during this developmental period. Nearly half of participants reported engaging in perpetrating unwanted sexual petting (49%), with substantial proportions also reporting unwanted oral (36%), vaginal (32%), and anal sex (27%). Among those who were sexually active, many reported multiple partners, inconsistent condom use, and low perceived HIV risk. These descriptive findings highlight both the magnitude of risk and ongoing gaps in risk perception and actual risk and reinforcing the importance of combining sexual violence and HIV prevention within the same intervention framework.

Consistent with social norms theory, our data revealed significant misperceptions around violence-related attitudes. Adolescents tended to overestimate the extent to which peers condoned coercive or gender-inequitable behaviors, despite most peers expressing prosocial views. This pluralistic ignorance provided a clear rationale for a corrective norms strategy to facilitate movement of behaviors towards prevention of violence. Accordingly, we designed a poster campaign to make positive peer norms and behaviors more visible and salient, aiming to shift perceived peer approval and reduce conformity to harmful norms.

In contrast, norms and behaviors related to HIV prevention were largely aligned. Reported condom use was relatively high and accurately perceived by peers. Although we did not test whether aligned norms and behaviors reinforce one another, this pattern is consistent with the IMB model, which suggests that sustained protective behavior depends on both motivation and skills, alongside supportive norms (Fisher & Fisher, 2000). Guided by this interpretation, our classroom sessions focused on reinforcing behavioral skills—such as correct and consistent condom use, consent communication, and negotiation—while strengthening motivation through realistic risk appraisal, rather than on normative correction.

Finally, we identified accurately perceived yet harmful norms around consent-related behaviors and gender equity. Because these norms could not be addressed through misperception correction, they were integrated into the curriculum sessions through guided discussion and skill-building exercises. Activities focused on respectful sexual decision-making, clear communication within relationships, and bystander intervention strategies. The prevalence of sexual violence perpetration and multiple sexual partnerships further justified this skills-based focus. Together, these findings highlight two complimentary intervention pathways: correcting misperceived norms around sexual violence and building motivation and behavioral skills where harmful norms are accurately perceived.

### Theoretical and practical implications

This integrated approach—pairing normative correction with behavioral skills training—offers complementary mechanisms for preventing sexual violence and HIV. For sexual violence prevention, addressing misperceptions around coercion, gender roles, and bystander action may shift the perceived social environment in ways that promote prosocial behavior (Berkowitz, 2003; Cialdini & Trost, 1998). For HIV prevention, where norms and behaviors are already aligned, emphasis on skill reinforcement and motivation may be more effective (Pettifor et al., 2015; Toska et al., 2017). Together, these findings suggest that normative and behavioral pathways work in tandem and that integrating both may yield more sustainable change than relying on either alone.

Our work also extends existing theoretical models that have dominated prevention research in southern Africa. While gender-power frameworks (e.g. *Stepping* Stones) have been instrumental in addressing structural inequalities, our approach contributes an additional theoretical layer by integrating social norms correction with behavioral skill-building. This combination allows for both environmental and individual change: correcting peer misperceptions to reshape the social context, while equipping adolescents with communication and negotiation skills to act within that context. These theoretical linkages provide an expanded foundation for intervention design and evaluation in adolescent health.

The school setting offers a particularly promising platform for this dual approach. Schools serve as micro-communities where peer influence is central to identity formation and behavioral learning. Intervening at this level can simultaneously target both individual competencies and the broader normative climate that sustains risk behaviors (Michau, 2007, Raising Voices, 2008, 2018). By embedding the intervention in this ecology —through peer-visible messaging and participatory sessions—we aimed to shift norms and skills concurrently, thereby maximizing potential diffusion and sustainability of behavior change.

### Strengths and limitations

Key strengths of this study include its theoretically grounded, data-driven approach to developing an integrated intervention addressing both sexual violence and HIV. The inclusion of peers outside the target audience recognizes that social norms are shaped within broader networks, not just within homogenous groups of adolescent boys. The use of a large, diverse adolescent sample across three schools enhances generalizability and ecological validity. Importantly, our design moves beyond cognitive change alone: evidence suggests that interventions focused solely on attitudes and beliefs are insufficient to reduce perpetration (Porat et al., 2024), and must be accompanied by interventions that integrate strong behavioral change theory elements (Jewkes et al., 2021; UN Women, 2021). By coupling normative correction with in-person practice of consent, negotiation and bystander skills, our approach bridges the gap between awareness and action.

Nevertheless, limitations must be acknowledged. The cross-sectional design precludes causal inference, and self-reported measures —though administered anonymously via ACASI —may still introduce social desirability bias and underreporting. While we analyzed data from adolescents of all genders, we did not disaggregate perceived norms by gender in this analysis, as our aim was to capture the broader peer environment that shapes boys’ attitudes and behaviors. Future analyzes should examine gender-specific and subgroup variations in perceived norms to refine targeting. Finally, longitudinal evaluation will be needed to determine whether integrating normative and skills-based strategies produces additive or synergistic effects.

## Conclusion

Our findings demonstrate the promise of combining social norms correction with behavioral skills reinforcement to address the intertwined epidemics of sexual violence and HIV among adolescents. By grounding intervention design in empirical data and behavioral theory, this approach leverages both peer influence and individual agency within the school environment. Future research should assess the acceptability, feasibility and efficacy of this integrated model, including its potential to diffuse positive norms across peer networks and sustain change over time. Expanding theoretically grounded, contextually responsive interventions of this kind is critical to reducing the dual burdens of sexual violence and HIV among adolescents in South Africa and similar settings.

## Figures and Tables

**Table 1.** Social ecological survey Sociodemographic and Behavioral Characteristics.

Variable	Total sample *N* = 1431
**School**	
Secondary school 1	425 (30%)
Secondary school 2	514 (36%)
Secondary school 3	482 (34%)
**Age**	
13–14 years	27 (2%)
15 years	250 (18%)
16 years	489 (34%)
17 years	384 (27%)
18–19 years	274 (19%)
**Self-identified Gender**	
Female	891 (62%)
Male	525 (37%)
Transgender or non-binary	13 (1%)
**Self-identified ‘Race’^a^**	
Black African	1394 (98%)
Colored	5 (0.4%)
Other	21 (1%)
**Sexual Orientation**	
Heterosexual	863 (62%)
Bisexual	279 (20%)
Gay or lesbian	144 (10%)
Queer	90 (6%)
**Language spoken at home**	
IsiXhosa	1355 (95%)
Other	68 (5%)
**Vaginal sexual debut age**	
≤12 years	136 (16%)
13 years	88 (10%)
14 years	140 (17%)
15 years	202 (24%)
16 years	156 (19%)
17–18 years	78 (9%)
**Lifetime Sexual Activity by Type**	
Heavy petting	499 (36%)
Oral	558 (41%)
Anal	405 (30%)
Vaginal	850 (62%)
**Sexual Partners Lifetime**	
1–3 partners	573 (71%)
3 + partners	231 (29%)
**Ever Used a Condom^b^**	
Yes	631 (76%)
No	203 (24%)
**Sexual Violence Perpetration: Completed one or more of these acts during past 12 months**	
Heavy Petting	689 (49%)
Oral sex	500 (36%)
Vaginal sex	448 (32%)
Anal sex	382 (27%)
**Sexual Violence Perpetration: Attempted one or more of these acts during past 12 months**	
Oral	421 (30%)
Vaginal	123 (13%)
Anal	102 (10%)

Note: Totals may not sum to 100% or the full sample size due to missing data, ‘prefer not to answer’ responses, or items not applicable to all participants.

a Race is self-identified, and we use categories as defined in the South African census. We see race as a social construct. The apartheid system coined the categories of ‘Black’ and ‘Colored’ to achieve racial stratification needed to effect discriminatory practice. The use of these terms here does not legitimize their validity or biological plausibility but is rather intended to foreground the way discrimination was deployed by previous governments to determine health opportunities for all South Africa – on a discriminatory basis.

b Male and female condom included in question.

## Data Availability

The data that support the findings of this study are available from the Principal Investigators of the study, C.K., C.M., A.H., upon reasonable request.

## Appendices

### Appendix B

**Table 2.** Individual variables used to calculate level of misperception.

Measure	Individual Norm	Individual Norm Response Options	Perceived Norm	Perceived Norm Response Options
Perception of Student Social Norms & Individual Student Social Norms	It is not OK to pressure someone to have sex	Strongly agree, Agree, Disagree, Strongly disagree	What percent of students at your school think it is OK to pressure someone to have sex?	0–100%
	It bothers me if a guy has sex with a girl who has had a lot of alcohol to drink.		What percent of students at your school are NOT bothered if a guy has sex with a girl who has had a lot of alcohol to drink?	
	It’s not OK to forward a naked picture of another student.		What percent of students at your school think it is OK to forward a naked picture of another student?	
	It’s not OK to give someone alcohol to make it easier to have sex with them.		What percent of students at your school think it is OK to give someone alcohol to make it easier to have sex with them?	
	I think it’s cool to brag about having lots of sexual partners.		What percent of students at your school think it’s cool to brag about having lots of sexual partners?	
	***I would respect someone who tries to stop sexual violence. (Sexual violence includes any unwanted or forced sexual activity/behavior).***		***What percent of students at your school would respect someone who tries to stop sexual violence? (Sexual violence includes any unwanted or forced sexual activity/behavior).***	
	***I would believe someone who says they were sexually assaulted.***		***What percent of students at your school would believe someone who says they were sexually assaulted?***	
Inequitable gender norms	Men need sex more than women do.	Disagree (Do not agree), Agree (Partially agree, Agree)	Most boys my age at my school believe men need sex more than women do.	Disagree (Do not agree), Agree (Partially agree, Agree)
	You don’t talk about sex, you just do it.		Most boys my age at my school believe you don’t talk about sex, you just do it.	
	Women who carry condoms on them are ‘easy.’		Most boys my age at my school believe women who carry condoms on them are ‘easy.’	
	***A man needs other women, even if things with his wife are fine.***		***Most boys my age at my school believe a man needs other women, even if things with his wife are fine.***	
	***There are times when a woman deserves to be beaten.***		***Most boys my age at my school believe there are times when a woman deserves to be beaten.***	
	It is a woman’s responsibility to avoid getting pregnant.		Most boys my age at my school believe it is a woman’s responsibility to avoid getting pregnant.	
	Men are always ready to have sex.		Most boys my age at my school believe men are always ready to have sex.	
	A woman should tolerate violence in order to keep her family together.		Most boys my age at my school believe a woman should tolerate violence in order to keep her family together.	
	***If a woman cheats on a man, it is okay for him to hit her.***		***Most boys my age at my school believe if a woman cheats on a man, it is okay for him to hit her.***	
	I would be outraged if my partner asked me to use a condom		For most boys my age at my school, he would be outraged if his wife asked him to use a condom.	
Equitable Gender Norms	A man should know what his partner likes during sex.	Do not agree, Partially agree, Agree	Most boys my age at my school believe a man should know what his partner likes during sex.	Do not agree, Partially agree, Agree
Bystander Attitude Scale	How likely would you be to stop sexual activity when asked to, even if you are already sexually aroused?	Not Likely (Not at all likely & Slightly likely), Likely (Somewhat likely, Likely, Moderately likely, Very likely)	How likely would MOST girls at your school be to stop sexual activity when asked to, even if they are already sexually aroused?	Not Likely (Not at all likely & Slightly likely), Likely (Somewhat likely, Likely, Moderately likely, Very likely)
	How likely would you be to stop sexual activity when asked to, even if you are already sexually aroused?		How likely would MOST boys at your school be to stop sexual activity when asked to, even if they are already sexually aroused?	
	How likely would you be to stop sexual activity when asked to, even if you are already sexually aroused?		How likely would MOST students at your school be to stop sexual activity when asked to, even if they are already sexually aroused?	
	How likely would you be to stop a sexual activity with someone if they say to stop, even if they were OK with it when it started?		How likely would MOST students at your school be to stop sexual activity with someone if they say to stop, even if they were OK with it when it started?	
	How likely would you be to decide not to have sexual activity with someone who is drunk?		How likely would MOST students at your school be to decide not to have sexual activity with someone who is drunk?	
	How likely would you be to ask for verbal consent when you are sexual with someone, even if you are in a long‐term relationship? (Verbal consent means to ask that a sexual activity is OK, before starting it).		How likely would MOST boys at your school be to ask for verbal consent when they are sexual with a girl, even if they were in a long-term relationship?	
	How likely would you be to ask for verbal consent when you are sexual with a guy, even if you are in a long‐term relationship? (Verbal consent means to ask that a sexual activity is OK, before starting it).		How likely would MOST girls at your school be to ask for verbal consent when they are sexual with a guy, even if they were in a long-term relationship?	
	How likely would you be to ask for verbal consent when you are sexual with someone, even if you are in a long‐term relationship? (Verbal consent means to ask that a sexual activity is OK, before starting it).		How likely would MOST students at your school be to ask for verbal consent when they are sexual with someone, even if they were in a long-term relationship?	
Perceptions of School Personnel	I would do something (for example, pull the kid back who was being aggressive) during a physical fight between a couple who is dating (or get school staff who could do this).		Most girls at my school would do something (for example, pull the kid back who was being aggressive) during a physical fight between a couple who is dating (or get school staff who could do this)	
	I would do something (for example, pull the kid back who was being aggressive) during a physical fight between a couple who is dating (or get school staff who could do this).		Most boys at my school would do something (for example, pull the kid back who was being aggressive) during a physical fight between a couple who is dating (or get school staff who could do this)	
Perception of Student Social Norms & Individual Student Social Norms	***I want a teacher to stop students from making unwanted sexual comments or jokes.***		***What percent of students at your school want a teacher to stop students from making unwanted sexual comments or jokes?***	
	***I believe that bystanders can take steps to prevent sexual violence. (A bystander is someone who sees something happen).***		***What percent of students at your school believe that bystanders can take steps to prevent sexual violence? (A bystander is someone who sees something happening).***	
	***I believe that bystanders can take steps to prevent sexual violence. (A bystander is someone who sees something happen).***		***What percent of girls at your school believe that bystanders can take steps to prevent sexual violence?***	
	***I believe that bystanders can take steps to prevent sexual violence. (A bystander is someone who sees something happen).***		***What percent of boys at your school believe that bystanders can take steps to prevent sexual violence?***	
HIV	In my opinion, a woman can suggest using condoms just like a man can.		Most boys my age at my school believe a woman can suggest using condoms just like a man can.	

Note 1: Table 2 presents only the variables shortlisted for misperceived norm calculations, identified through formative work with similar populations in South Africa. The full measures with all variables are provided in Table 3. The table is formatted to display each individual norm alongside the corresponding perceived peer norm used in this calculation.

Note 2: Variables that are both bolded and italicized indicate those that were significantly misperceived—defined as a greater than 20% difference between the individual norm and the perceived peer norm—across all three secondary schools.

### Appendix C

**Table 3.** Supplementary table of full measures.

Measurement name & citation	Question	Response options
Sexual Experiences Survey (SES)- Short Form Koss, M. P., Abbey, A., Campbell, R., Cook, S., Norris, J., Testa, M.,. & White, J. (2007). Revising the SES: A collaborative process to improve assessment of sexual aggression and victimization. Psychology of Women Quarterly, 31(4), 357–370. https://doi.org/10.1111/j.1471–6402.2007.00385.x	How many times in the past 12 months did you fondle, kiss, or rub up against the private areas of someone’s body (lips, breast/chest, crotch or butt) or remove some of their clothes when they did not want this?	<0> 0 times <1> 1 time <2> 2 times <3> 3 or more times
	Was this by? [Select all that apply]	<1> Telling lies, threatening to end the relationship, threatening to spread rumors about them, making promises about the future you knew were untrue, or continually verbally pressuring them after they said they didn’t want to. <2> Showing displeasure, criticizing their sexuality or attractiveness, getting angry but not using physical force after they said they didn’t want to. <3> Taking advantage when they were too drunk or out of it to stop what was happening. <4> Threatening to physically harm them or someone close to them. <5> Using force, for example holding them down with your body weight, pinning their arms, or having a weapon.
	How many times in the past 12 months have you had oral sex with someone or had someone perform oral sex on you when they did not want this?	<0> 0 times <1> 1 time <2> 2 times <3> 3 or more times
	Was this by? [Select all that apply]	<1> Telling lies, threatening to end the relationship, threatening to spread rumors about them, making promises about the future you knew were untrue, or continually verbally pressuring them after they said they didn’t want to. <2> Showing displeasure, criticizing their sexuality or attractiveness, getting angry but not using physical force after they said they didn’t want to. <3> Taking advantage when they were too drunk or out of it to stop what was happening. <4> Threatening to physically harm them or someone close to them. <5> Using force, for example holding them down with your body weight, pinning their arms, or having a weapon.
	How many times in the past 12 months have you put your fingers or objects into a woman’s vagina when she did not want this?	<0> 0 times <1> 1 time <2> 2 times <3> 3 or more times
	Was this by? [Select all that apply]	<1> Telling lies, threatening to end the relationship, threatening to spread rumors about them, making promises about the future you knew were untrue, or continually verbally pressuring them after they said they didn’t want to. <2> Showing displeasure, criticizing their sexuality or attractiveness, getting angry but not using physical force after they said they didn’t want to. <3> Taking advantage when they were too drunk or out of it to stop what was happening. <4> Threatening to physically harm them or someone close to them. <5> Using force, for example holding them down with your body weight, pinning their arms, or having a weapon.
	How many times in the past 12 months have you put your fingers or objects into someone’s butt if they did not want this?	<0> 0 times <1> 1 time <2> 2 times <3> 3 or more times
	Was this by? [Select all that apply]	<1> Telling lies, threatening to end the relationship, threatening to spread rumors about them, making promises about the future you knew were untrue, or continually verbally pressuring them after they said they didn’t want to. <2> Showing displeasure, criticizing their sexuality or attractiveness, getting angry but not using physical force after they said they didn’t want to. <3> Taking advantage when they were too drunk or out of it to stop what was happening. <4> Threatening to physically harm them or someone close to them. <5> Using force, for example holding them down with your body weight, pinning their arms, or having a weapon.
	Even though it did not happen, how many times in the past 12 months did you TRY to have oral sex with someone when they did not want to?	<0> 0 times <1> 1 time <2> 2 times <3> 3 or more times
	Was this by? [Select all that apply]	<1> Telling lies, threatening to end the relationship, threatening to spread rumors about them, making promises about the future you knew were untrue, or continually verbally pressuring them after they said they didn’t want to. <2> Showing displeasure, criticizing their sexuality or attractiveness, getting angry but not using physical force after they said they didn’t want to. <3> Taking advantage when they were too drunk or out of it to stop what was happening. <4> Threatening to physically harm them or someone close to them. <5> Using force, for example holding them down with your body weight, pinning their arms, or having a weapon.
	Even though it did not happen, how many times in the past 12 months did you TRY to put your fingers or objects into a woman’s vagina when they did not want this?	<0> 0 times <1> 1 time <2> 2 times <3> 3 or more times
	Was this by? [Select all that apply]	<1> Telling lies, threatening to end the relationship, threatening to spread rumors about them, making promises about the future you knew were untrue, or continually verbally pressuring them after they said they didn’t want to. <2> Showing displeasure, criticizing their sexuality or attractiveness, getting angry but not using physical force after they said they didn’t want to. <3> Taking advantage when they were too drunk or out of it to stop what was happening. <4> Threatening to physically harm them or someone close to them. <5> Using force, for example holding them down with your body weight, pinning their arms, or having a weapon.
	Even though it did not happen, how many times in the past 12 months did you TRY to put your fingers or objects into someone’s butt when they did not want this?	<0> 0 times <1> 1 time <2> 2 times <3> 3 or more times
	Was this by? [Select all that apply]	<1> Telling lies, threatening to end the relationship, threatening to spread rumors about them, making promises about the future you knew were untrue, or continually verbally pressuring them after they said they didn’t want to. <2> Showing displeasure, criticizing their sexuality or attractiveness, getting angry but not using physical force after they said they didn’t want to. <3> Taking advantage when they were too drunk or out of it to stop what was happening. <4> Threatening to physically harm them or someone close to them. <5> Using force, for example holding them down with your body weight, pinning their arms, or having a weapon.
	Did you do any of these acts 1 or more times?	<1> Yes <0> No
	Do you think you may have ever raped someone?	<1> Yes <0> No
	Were you using alcohol or other drugs during these acts?	<1> Alcohol <2> Drugs <3> Both <4> None
	Was the other person(s) using alcohol or other drugs during these acts?	<1> Alcohol <2> Drugs <3> Both <4> None
The Sexual Social Norms Inventory Bruner, J. B. (2002). Measuring rape-supportive attitudes, behaviors, and perceived peer norms among college students: Validation of a social norms survey (Unpublished doctoral dissertation). University of Northern Colorado, Greeley.	These questions are about your OWN beliefs. [INDSOCNORM01] It is not OK to pressure someone to have sex.	<1> Strongly Disagree <2> Disagree <3> Agree <4> Strongly Agree
	[INDSOCNORM02] I am bothered when teens use the word ‘gay’ or ‘lesbian’ in a mean way.	<1> Strongly Disagree <2> Disagree <3> Agree <4> Strongly Agree
	[INDSOCNORM03] It is not a big deal if someone is bullied. (Bullying includes threatening, spreading rumors, hitting, shoving or hurting someone in person, online, through email, text or social media).	<1> Strongly Disagree <2> Disagree <3> Agree <4> Strongly Agree
	[INDSOCNORM04] It bothers me if a guy has sex with a girl who has had a lot of alcohol to drink.	<1> Strongly Disagree <2> Disagree <3> Agree <4> Strongly Agree
	[INDSOCNORM05] It’s not OK to forward a naked picture of another student.	<1> Strongly Disagree <2> Disagree <3> Agree <4> Strongly Agree
	[INDSOCNORM06] It’s not OK to give someone alcohol to make it easier to have sex with them.	<1> Strongly Disagree <2> Disagree <3> Agree <4> Strongly Agree
	[INDSOCNORM07] I think it’s cool to brag about having lots of sexual partners.	<1> Strongly Disagree <2> Disagree <3> Agree <4> Strongly Agree
	[INDSOCNORM08] I would respect someone who tries to stop sexual violence. (Sexual violence includes any unwanted or forced sexual activity/behavior).	<1> Strongly Disagree <2> Disagree <3> Agree <4> Strongly Agree
	[INDSOCNORM09] I want a teacher to stop students from making unwanted sexual comments or jokes.	<1> Strongly Disagree <2> Disagree <3> Agree <4> Strongly Agree
	[INDSOCNORM10] I believe that bystanders can take steps to prevent sexual violence. (A bystander is someone who sees something happen).	<1> Strongly Disagree <2> Disagree <3> Agree <4> Strongly Agree
	[INDSOCNORM11] I would believe someone who says they were sexually assaulted.	<1> Strongly Disagree <2> Disagree <3> Agree <4> Strongly Agree
The Bystander Intervention subscale of the Sexual Social Norms Inventory Bruner, J. B. (2002). Measuring rape-supportive attitudes, behaviors, and perceived peer norms among college students: Validation of a social norms survey (Unpublished doctoral dissertation). University of Northern Colorado, Greeley.	Please indicate how likely you are to engage in each of the following: Sexual activity includes any kind of sexual behavior (kissing, touching, or sexual intercourse).	
	[BYSTANATT01] How likely would you be to stop sexual activity when asked to, even if you are already sexually aroused?	<1> Not at all likely <2> Slightly likely <3> Somewhat likely <4> Likely <5> Moderately likely <6> Very likely
	[BYSTANATT02] How likely would MOST girls at your school be to stop sexual activity when asked to, even if they are already sexually aroused?	<1> Not at all likely <2> Slightly likely <3> Somewhat likely <4> Likely <5> Moderately likely <6> Very likely
	[BYSTANATT03] How likely would MOST boys at your school be to stop sexual activity when asked to, even if they are already sexually aroused?	<1> Not at all likely <2> Slightly likely <3> Somewhat likely <4> Likely <5> Moderately likely <6> Very likely
	[BYSTANATT04] How likely would MOST students at your school be to stop sexual activity when asked to, even if they are already sexually aroused?	<1> Not at all likely <2> Slightly likely <3> Somewhat likely <4> Likely <5> Moderately likely <6> Very likely
	[BYSTANATT05] How likely would you be to stop a sexual activity with someone if they say to stop, even if they were OK with it when it started?	<1> Not at all likely <2> Slightly likely <3> Somewhat likely <4> Likely <5> Moderately likely <6> Very likely
	[BYSTANATT06] How likely would MOST students at your school be to stop sexual activity with someone if they say to stop, even if they were OK with it when it started?	<1> Not at all likely <2> Slightly likely <3> Somewhat likely <4> Likely <5> Moderately likely <6> Very likely
	[BYSTANATT07] How likely would you be to decide not to have sexual activity with someone who is drunk?	<1> Not at all likely <2> Slightly likely <3> Somewhat likely <4> Likely <5> Moderately likely <6> Very likely
	[BYSTANATT08] How likely would MOST students at your school be to decide not to have sexual activity with someone who is drunk?	<1> Not at all likely <2> Slightly likely <3> Somewhat likely <4> Likely <5> Moderately likely <6> Very likely
	[BYSTANATT09] How likely would you be to ask for verbal consent when you are sexual with someone, even if you are in a long‐term relationship? (Verbal consent means to ask that a sexual activity is OK, before starting it).	<1> Not at all likely <2> Slightly likely <3> Somewhat likely <4> Likely <5> Moderately likely <6> Very likely
	[BYSTANATT10] How likely would MOST students at your school be to ask for verbal consent when they are sexual with someone, even if they were in a long-term relationship?	<1> Not at all likely <2> Slightly likely <3> Somewhat likely <4> Likely <5> Moderately likely <6> Very likely
	[BYSTANATT11] How likely would you be to ask for verbal consent when you are sexual with a guy, even if you are in a long‐term relationship? (Verbal consent means to ask that a sexual activity is OK, before starting it).	<1> Not at all likely <2> Slightly likely <3> Somewhat likely <4> Likely <5> Moderately likely <6> Very likely
	[BYSTANATT12] How likely would MOST girls at your school be to ask for verbal consent when they are sexual with a guy, even if they were in a long-term relationship?	<1> Not at all likely <2> Slightly likely <3> Somewhat likely <4> Likely <5> Moderately likely <6> Very likely
	[BYSTANATT13] How likely would MOST boys at your school be to ask for verbal consent when they are sexual with a girl, even if they were in a long-term relationship?	<1> Not at all likely <2> Slightly likely <3> Somewhat likely <4> Likely <5> Moderately likely <6> Very likely
Illinois Rape Myth Acceptance Scale Cook-Craig, *P*. G., Coker, A. L., Clear, E. R., Garcia, L. S., Bush, H. M., Brancato, C. J.,. & Fisher, B. S. (2014). Challenge and opportunity in evaluating a diffusion-based active bystanding prevention program: Green Dot in high schools. Violence against women, 20(10), 1179–1202.	Please fill in the box that describes how much you agree or disagree with the following statements.	
	[GENDERNORM01] Girls should have sex with their boyfriend or the guy they are dating when he wants.	<1> Strongly Disagree <2> Disagree <3> Agree <4> Strongly Agree
	[GENDERNORM02] If a guy spends money on a date, the girl should have sex with him in return.	<1> Strongly Disagree <2> Disagree <3> Agree <4> Strongly Agree
	[GENDERNORM03] Guys should respond to dates’ or girlfriends’ challenges to authority by insulting them or putting them down.	<1> Strongly Disagree <2> Disagree <3> Agree <4> Strongly Agree
	[GENDERNORM04] If a girl is sexually assaulted while she is drunk, she is to blame for letting things get out of control.	<1> Strongly Disagree <2> Disagree <3> Agree <4> Strongly Agree
	[GENDERNORM05] Sexual assault charges are often used as a way of getting back at guys.	<1> Strongly Disagree <2> Disagree <3> Agree <4> Strongly Agree
	[GENDERNORM06] Many girls lead a guy on, and then they claim it was sexual assault.	<1> Strongly Disagree <2> Disagree <3> Agree <4> Strongly Agree
	[GENDERNORM07] When girls are sexually assaulted, it is often because the way they said ‘no’ was unclear.	<1> Strongly Disagree <2> Disagree <3> Agree <4> Strongly Agree
The Differential Reinforcement subscale of the Social Norms Measure Boeringer, S. B., Shehan, C. L., & Akers, R. L. (1991). Social contexts and social learning in sexual coercion and aggression: Assessing the contribution of fraternity membership. Family Relations, 58–64. https://doi.org/10.2307/585659	[SOCNORM01] How many of your friends have gotten a woman drunk or high in order to have sex with her?	<1> None <2> One or two <3> Three to five <4> Six to ten <5> More than ten
	[SOCNORM02] How many of your friends have forced or tried to force sex on a woman, such as a ‘known tease,’ who refused to have sex?	<1> None <2> One or two <3> Three to five <4> Six to ten <5> More than ten
	How approving do you think your friends would be of you in the following circumstances:	
	[SOCNOMR03] If you had sexual intercourse with many women during the year?	<1> Very approving <2> Approving <3> Neutral <4> Disapproving <5> Very disapproving
	[SOCNOMR04] If you got a woman drunk or high in order to have sex with her?	<1> Very approving <2> Approving <3> Neutral <4> Disapproving <5> Very disapproving
	[SOCNOMR05] If you forced a ‘known tease’ to have sex with you, after she had teased you and then refused to have sex?	<1> Very approving <2> Approving <3> Neutral <4> Disapproving <5> Very disapproving
	If you were to engage in any of the following acts, do you anticipate that the experience would be mainly pleasurable or rewarding to you, mainly negative or unpleasant, or somewhere in between?	
	[SOCNORM06] Forcing a female to do something sexual she didn’t want to do.	<1> Mainly pleasurable or rewarding <2> Mainly negative or unpleasant <3> Somewhere in between
	[SOCNORM07] Rape.	<1> Mainly pleasurable or rewarding <2> Mainly negative or unpleasant <3> Somewhere in between
	Nearly all men have at one point or another viewed so-called ‘men’s’ magazines, which depict nude females. Please indicate the number of times you have viewed these kinds of material, as well as the other kinds of material listed below in your lifetime.	
	[SOCNORM08] Magazines which depict a female or females being forced to engage in sexual acts.	<1> Never <2> 1 to 5 times <3> 6 to 10 times <4> 11 to 20 times <5> More than 20 times
	[SOCNORM09] Videos or films which depict a female or females being forced to engage in sexual acts.	<1> Never <2> 1 to 5 times <3> 6 to 10 times <4> 11 to 20 times <5> More than 20 times
	[SOCNORM10] Written books, without photos, which depict a female or females being forced to engage in sexual acts.	<1> Never <2> 1 to 5 times <3> 6 to 10 times <4> 11 to 20 times <5> More than 20 times
	If you could be assured that you could in no way be punished for engaging in the following acts, how likely, if at all, would you be to engage in such acts?	
	[SOCNORM11] Forcing a female to do something sexual she didn’t want to do.	<1> Very unlikely <2> Unlikely <3> Neutral <4> Likely <5> Very likely
	[SOCNORM12] Rape.	<1> Very unlikely <2> Unlikely <3> Neutral <4> Likely <5> Very likely
The Gender-Equitable Men Scale Pulerwitz, J., & Barker, G. (2008). Measuring attitudes toward gender norms among young men in Brazil: development and psychometric evaluation of the GEM scale. Men and masculinities, 10(3), 322–338.	These questions ask about your OWN beliefs and then about the beliefs of BOYS YOUR AGE.	
	[INGNDRNORM01] It is the man who decides what type of sex to have.	<1> Do not agree <2> Partially agree <3> Agree
	[INGNDRNORM02] Most boys my age at my school believe it is the man who decides what type of sex to have.	<1> Do not agree <2> Partially agree <3> Agree
	[INGNDRNORM03] A woman’s most important role is to take care of her home and cook for her family.	<1> Do not agree <2> Partially agree <3> Agree
	[INGNDRNORM04] Most boys my age at my school believe a woman’s most important role is to take care of her home and cook for her family.	<1> Do not agree <2> Partially agree <3> Agree
	[INGNDRNORM05] Men need sex more than women do.	<1> Do not agree <2> Partially agree <3> Agree
	[INGNDRNORM06] Most boys my age at my school believe men need sex more than women do.	<1> Do not agree <2> Partially agree <3> Agree
	[INGNDRNORM07] You don’t talk about sex, you just do it.	<1> Do not agree <2> Partially agree <3> Agree
	[INGNDRNORM08] Most boys my age at my school believe you don’t talk about sex, you just do it.	<1> Do not agree <2> Partially agree <3> Agree
	[INGNDRNORM09] Women who carry condoms on them are ‘easy.’	<1> Do not agree <2> Partially agree <3> Agree
	[INGNDRNORM10] Most boys my age at my school believe women who carry condoms on them are ‘easy.’	<1> Do not agree <2> Partially agree <3> Agree
	[INGNDRNORM11] A man needs other women, even if things with his wife are fine.	<1> Do not agree <2> Partially agree <3> Agree
	[INGNDRNORM12] Most boys my age at my school believe a man needs other women, even if things with his wife are fine.	<1> Do not agree <2> Partially agree <3> Agree
	[INGNDRNORM13] There are times when a woman deserves to be beaten.	<1> Do not agree <2> Partially agree <3> Agree
	[INGNDRNORM14] Most boys my age at my school believe there are times when a woman deserves to be beaten.	<1> Do not agree <2> Partially agree <3> Agree
	[INGNDRNORM15] Changing diapers/nappies, giving the kids a bath, and feeding the kids are the mother’s responsibility.	<1> Do not agree <2> Partially agree <3> Agree
	[INGNDRNORM16] Most boys my age at my school believe changing diapers/nappies, giving the kids a bath, and feeding the kids are the mother’s responsibility.	<1> Do not agree <2> Partially agree <3> Agree
	[INGNDRNORM17] It is a woman’s responsibility to avoid getting pregnant.	<1> Do not agree <2> Partially agree <3> Agree
	[INGNDRNORM18] Most boys my age at my school believe it is a woman’s responsibility to avoid getting pregnant.	<1> Do not agree <2> Partially agree <3> Agree
	[INGNDRNORM19] A man should have the final word about decisions in his home.	<1> Do not agree <2> Partially agree <3> Agree
	[INGNDRNORM20] Most boys my age at my school believe a man should have the final word about decisions in his home.	<1> Do not agree <2> Partially agree <3> Agree
	[INGNDRNORM21] Men are always ready to have sex.	<1> Do not agree <2> Partially agree <3> Agree
	[INGNDRNORM22] Most boys my age at my school believe men are always ready to have sex.	<1> Do not agree <2> Partially agree <3> Agree
	[INGNDRNORM23] A woman should tolerate violence in order to keep her family together.	<1> Do not agree <2> Partially agree <3> Agree
	[INGNDRNORM24] Most boys my age at my school believe a woman should tolerate violence in order to keep her family together.	<1> Do not agree <2> Partially agree <3> Agree
	[INGNDRNORM25] If a woman cheats on a man, it is okay for him to hit her.	<1> Do not agree <2> Partially agree <3> Agree
	[INGNDRNORM26] Most boys my age at my school believe if a woman cheats on a man, it is okay for him to hit her.	<1> Do not agree <2> Partially agree <3> Agree
	[INGNDRNORM27] If someone insults me, I will defend my reputation, with force if I have to.	<1> Do not agree <2> Partially agree <3> Agree
	[INGNDRNORM28] For most boys my age at my school, if someone insults them, they will defend their reputation with force if they have to.	<1> Do not agree <2> Partially agree <3> Agree
	[INGNDRNORM29] I would be outraged if my partner asked me to use a condom.	<1> Do not agree <2> Partially agree <3> Agree
	[INGNDRNORM30] For most boys my age at my school, he would be outraged if his wife asked him to use a condom.	<1> Do not agree <2> Partially agree <3> Agree
	[INGNDRNORM31] It is okay for a man to hit his wife if she won’t have sex with him.	<1> Do not agree <2> Partially agree <3> Agree
	[INGNDRNORM32] Most boys my age at my school believe it is okay for a man to hit his wife if she won’t have sex with him.	<1> Do not agree <2> Partially agree <3> Agree
	[INGNDRNORM33] I would never have a gay friend.	<1> Do not agree <2> Partially agree <3> Agree
	[INGNDRNORM34] Most boys my age at my school would never have a gay friend.	<1> Do not agree <2> Partially agree <3> Agree
	[EGNDRNORM01] A couple should decide together if they want to have children.	<1> Do not agree <2> Partially agree <3> Agree
	[EGNDRNORM02] Most boys my age at my school believe a couple should decide together if they want to have children.	<1> Do not agree <2> Partially agree <3> Agree
	[EGNDRNORM03] In my opinion, a woman can suggest using condoms just like a man can.	<1> Do not agree <2> Partially agree <3> Agree
	[EGNDRNORM04] Most boys my age at my school believe a woman can suggest using condoms just like a man can.	<1> Do not agree <2> Partially agree <3> Agree
	[EGNDRNORM05] If a guy gets a woman pregnant, the child is the responsibility of both.	<1> Do not agree <2> Partially agree <3> Agree
	[EGNDRNORM06] Most boys my age at my school believe if a guy gets a woman pregnant, the child is the responsibility of both.	<1> Do not agree <2> Partially agree <3> Agree
	[EGNDRNORM07] A man should know what his partner likes during sex.	<1> Do not agree <2> Partially agree <3> Agree
	[EGNDRNORM08] Most boys my age at my school believe a man should know what his partner likes during sex.	<1> Do not agree <2> Partially agree <3> Agree
	[EGNDRNORM09] It is important that a father is present in the lives of his children, even if he is no longer with the mother.	<1> Do not agree <2> Partially agree <3> Agree
	[EGNDRNORM10] Most boys my age at my school believe it is important that a father is present in the lives of his children, even if he is no longer with the mother.	<1> Do not agree <2> Partially agree <3> Agree
	[EGNDRNORM11] A man and a woman should decide together what type of contraceptive to use.	<1> Do not agree <2> Partially agree <3> Agree
	[EGNDRNORM12] Most boys at my school believe a man and a woman should decide together what type of contraceptive to use.	<1> Do not agree <2> Partially agree <3> Agree
	[EGNDRNORM13] It is important to have a male friend that you can talk about your problems with.	<1> Do not agree <2> Partially agree <3> Agree
	[EGNDRNORM14] Most boys at my school believe it is important to have a male friend that you can talk about your problems with.	<1> Do not agree <2> Partially agree <3> Agree
